# Involuntary reflexive pelvic floor muscle training in addition to standard training versus standard training alone for women with stress urinary incontinence: a randomized controlled trial

**DOI:** 10.1007/s00192-021-04701-5

**Published:** 2021-02-10

**Authors:** Helena Luginbuehl, Corinne Lehmann, Irene Koenig, Annette Kuhn, Reto Buergin, Lorenz Radlinger

**Affiliations:** 1 Department of Health Professions, Division of Physiotherapy, Bern University of Applied Sciences, Murtenstrasse 10, 3008 Bern, Switzerland; 2 Department of Physiotherapy, Bern University Hospital and University of Bern, Bern, Switzerland; 3 Faculty of Physical Education and Physiotherapy, Vrije Universiteit Brussel, Brussels, Belgium; 4 Urogynaecology, Bern University Hospital and University of Bern, Women’s Hospital, Bern, Switzerland; 5 Department of Health Professions, Division of Nursing, Bern University of Applied Sciences, Bern, Switzerland

**Keywords:** Exercise, Muscle contraction, Physical therapy modalities, Reflex

## Abstract

**Introduction and hypothesis:**

Although involuntary reflexive pelvic floor muscle contractions seem crucial during stress urinary incontinence-provoking situations, hitherto existing guidelines feature voluntary pelvic floor muscle training only. Two pelvic floor muscle protocols were compared regarding their effect on stress urinary incontinence in women: one focusing on standard physiotherapy with voluntary pelvic floor muscle training, the other additionally including involuntary reflexive pelvic floor muscle training.

**Methods:**

This study was designed as a triple-blind prospective randomized controlled trial with women suffering from stress urinary incontinence with two physiotherapy intervention groups (control group: standard physiotherapy, *n* = 48, experimental group: standard physiotherapy plus involuntary reflexive pelvic floor muscle training triggered by whole-body movements such as jumps *n* = 48). Both interventions lasted 16 weeks (9 personal physiotherapy consultations and 78 home training sessions). Group differences and development over time were analyzed concerning the primary outcome International Consultation on Incontinence Modular Questionnaire Urinary Incontinence short form (ICIQ-UIsf) by mixed effect regression models.

**Results:**

The ICIQ-UIsf score decreased significantly over time for both groups by about 3 points from about 10 to about 7 points with no group differences at any point in time.

**Discussion:**

This trial did not find any additional benefit for stress urinary incontinence by adding involuntary reflexive pelvic floor muscle training to standard training. Both training protocols showed similar clinically relevant improvements; however, there was still moderate incontinence after interventions. Future studies should test and apply pelvic floor muscle function-oriented training methods for pelvic floor muscle hypertrophy, intramuscular coordination, and power, which are more in line with conventional skeletal muscle training, i.e., performed with higher intensities and workout.

## Introduction

Women suffering from stress urinary incontinence (SUI) complain of urine loss during coughing, sneezing or physical exertion, i.e. sporting activities [1]. Thus, the core triggers of SUI are high load impacts [2] and high ground reaction forces within milliseconds [3], which cause high intra-abdominal pressure [4]. Therefore, fast involuntary reflexive pelvic floor muscle (PFM) contractions are required to guarantee continence [5]. Fast involuntary reflexive PFM contractions were observed during coughing [6], running [7], trampolining [8] and jumping [8, 9] within the time interval of 30 ms before (pre-activation) to 150 ms after an impact. The latter characterizes slow (30–60 ms), mid (60–90 ms), and long latency (90–120 ms) reflex responses and long latency succeeding (120–150 ms) reflex responses [10].

Although fast voluntary PFM contractions (time to peak: ~500 ms) in no way reach the PFM rate of activity or force development required for high-impact situations [11], PFM training (PFMT) has so far only been described based on voluntary PFM contractions [12, 13]. Training procedures following the concepts of training science and sports medicine are generally evident, well known and widely implemented in rehabilitation and sports [14]; however, an optimal and well-standardized training protocol that also includes involuntary reflexive PFM contractions remains unknown.

When the PFMs are contracted voluntarily and therefore concentrically, resulting in a cranial movement, there is no certain and specific load in the sense of concentric strength training, apart from the load of the internal organs and intra-abdominal pressure. However, many types of training require specific loads to achieve the intended training effects (sensorimotor effects, hypertrophy, intramuscular coordination, etc.). In a stretch-shortening cycle (SSC; i.e., eccentric–concentric contractions) during jumping, for instance, because of the rapid stretching of the tendon, connective tissue, and muscular system, a reflex is triggered and elastic energy is stored, which both flow into the immediately following concentric phase. In this way, a very high force is generated in a very short time period. This aspect has long been known and well investigated in exercise physiology [15]. However, this reactive muscle behavior has not yet been transferred to PFMT with rapid and supra-maximal involuntary and reflexive PFM activation, allowing for “power training.” Even if the eccentric–concentric SSC for the PFM has so far not been confirmed in investigations of exercises like running, counter movement jumps, drop jumps, and drop landings, very high and fast reflexive (pre-)activations of the PFM could be found [7–9].

To fill this gap in involuntary reflexive PFMT protocols, a standardized PFMT protocol including standard physiotherapy (PT; i.e., the standard PFMT of the authors’ unit, which is based on voluntary PFM contractions [12, 13] and basic information and instructions) and additionally focusing on involuntary fast reflexive PFM contractions, was developed [16]. The aim of the present trial was to compare this newly developed PFMT with a standard PFMT regarding their effect on SUI [16].

## Materials and methods

The detailed concept and methods, as well as the extensive standardized training protocol of this trial, are published in Luginbuehl et al. [16]. For this reason, only a summary is given here.

This investigation was designed as a triple-blind (participants, investigators, statistician) prospective randomized controlled trial with two PT intervention groups. This means that participants, investigators and the statistician had no knowledge of the group assignment of the participants. The randomization of group allocation (allocation ratio = 1:1 – control group: experimental group) followed the online tool www.randomization.com. The code and group allocation of each participant was packed into concealed opaque envelopes. The envelopes were stored at the urogynecology secretariat. Physiotherapists treating participants were informed about group allocation of their participant by the independent urogynecology secretariat. The study protocol was approved by the Ethics Committee of the Canton of Bern, Switzerland (reference number 249/14), registered at (www.clinicaltrials.gov; study identifier: NCT02318251), and published including the detailed intervention PFMT description as “Additional file” [16]. The trial was funded by the Swiss National Science Foundation, Division III (Medicine & Biology, No. 153424).

### Participants

For this study, 96 participants were consecutively included under the following predetermined conditions after giving written informed consent: women aged 18–70 years suffering from SUI (based on the participant’s history) or mixed urinary incontinence, but predominantly SUI. Further inclusion criteria were: at least 1 year post-partum, parous, nulliparous, pre- or post-menopausal, BMI of 18–30 kg/m^2^, medical and physical fitness for the therapeutic exercises (especially running and jumps), and, in the case of systemic or local estrogen treatment, being stable for the 3 months prior to inclusion. Exclusion criteria were urge incontinence or predominant urgency incontinence, prolapse > stage 1 POP-Q (uterus, anterior and posterior vaginal wall prolapse during straining maneuver), PFM strength grading of 0 (meaning no discernible muscle contraction) digitally assessed according to the Oxford Grading Scale [17], pregnancy (urine test to confirm), current urinary tract or vaginal infection, menstruation on the day of examination, lactation period not yet finished, contraindications for measurements or interventions (e.g., acute inflammatory or infectious disease, tumor, fracture), de novo systemic or local estrogen treatment (<3 months), de novo drug treatment with anticholinergics or other bladder active substances (tricyclic antidepressants, selective serotonin reuptake inhibitors) [16].

### Intervention

Both PFMT protocols were standardized and included basic information and instructions (e.g., performance of correct PFM contraction, information regarding anatomical and (patho-)physiological aspects of SUI, exercising pre-contraction, fluid intake, micturition and defecation behavior). These protocols were based on evident motor learning and strength training concepts with progression of training for strength, power and hypertrophy, and involved an intervention of 16 weeks, including nine personal PT consultations and 78 short home training sessions [16]. The main difference between the protocols is that in the control group (CON), isometric and concentric voluntary PFM contractions were performed in a slow to moderate to fast speed of movement; whereas in the experimental group (EXP), isometric as well as concentric pelvic floor muscle contractions were carried out voluntarily but explosively, and involuntary PFM contractions were caused by exercises such as running on the spot, counter-movement jumps, and drop jumps to result in an explosive, re-active, and reflexive speed of movement in terms of power training. “Power” has to be interpreted as mechanical power—P(t) = F x v: power equals force times velocity—in the sense of rate of force development (or rate of PFM activity development) in the context of power training described here, with fast or explosive voluntary or involuntary contractions. For the additional involuntary reflexive PFMT in the experimental group, whole body movements, which trigger PFM reflex activity [7–9] such as jumps were applied as very specific PFMT stimuli. Detailed information about the respected training principles is provided in the study protocol [16].

### Outcomes

Primary outcome was the International Consultation on Incontinence Modular Questionnaire Urinary Incontinence short form (ICIQ-UIsf). The ICIQ-UIsf is a validated patient-reported measure of severity (questions 3 and 4 [Q3 + Q4]) and impact of urinary incontinence on quality of life (question 5 [Q5]) in women [18]. It is scored for the total score on a scale from 0 (not) to 21 (severely affected) (subscore Q3 + Q4: 0–11; subscore Q5: 0–10). The ICIQ-UIsf score was collected 10 times: before (pre) and after (post) and every 2 weeks (week 2, 4, 6, 8, 10, 12, 14, and 16: PT2 … PT9) during the intervention phase.

Secondary outcomes were the ICIQ Lower Urinary Tract Symptoms Quality of Life Module (ICIQ-LUTSqol) [19] (Part A: 19–76 overall score with greater values indicating increased impact on quality of life, Part B: scores 0–200 bother scales are not incorporated in the overall score but indicate the impact of individual symptoms for the patient), a modified 20-min pad test, PFM strength (digitally assessed according to the Oxford Grading Scale (score 0–5) [17], and adherence to nine PT consultations and 78 home training sessions (number of individually accomplished training sessions) collected in a training diary. They were tested before and after the intervention phase.

### Sample size calculation

Sample size calculation was performed using G*Power software, using the statistical model of an ANOVA approach (repeated measures, within/between interactions). An effect size of = 0.1, indicating a small effect [20] was accepted. The sample size was calculated for the primary outcome ICIQ-UIsf with the following assumptions: α = 0.05, power = 0.8, groups = 2, measurements = 10, correlations = 0.5. Based on these assumptions, a total sample size of *N* = 80 was estimated. In anticipation of dropouts (*n* = 8) or a violation of normality assumption (*n* = 8), a final sample size of *N* = 96 (48 participants per group) was calculated. Anticipating that there will be dropouts during the intervention phase, the intention-to-treat analysis was chosen, and the last observation carried forward method was applied for imputing missing data in this longitudinal study. This means that if a participant drops out before the end of study, the last observed score of the dependent variable is used for all subsequent (i.e., missing) observation points.

### Statistics

All statistical calculations were performed using IBM SPSS 25 for Windows (SPSS, Chicago, IL, USA). Descriptive and analytical statistics for demographics and secondary outcomes were calculated according to the scaling of the data either parametrically (mean, standard deviation, independent and dependent sample *t* test) or nonparametrically (median, interquartile range, Wilcoxon signed rank test, Mann–Whitney *U* test). Mixed effect regression models were used for the primary outcome ICIQ-UIsf to analyze differences between the CON and the EXP groups, the recovery size, and the time to reach the bottom level. The implemented mixed models consist of fixed effects reflecting the average evolution of each group across time, and an autoregressive component of order 1 to consider for intra-participant correlation. The mixed models were estimated using the MIXED procedure in SPSS.

## Results

**Fig. 1:**
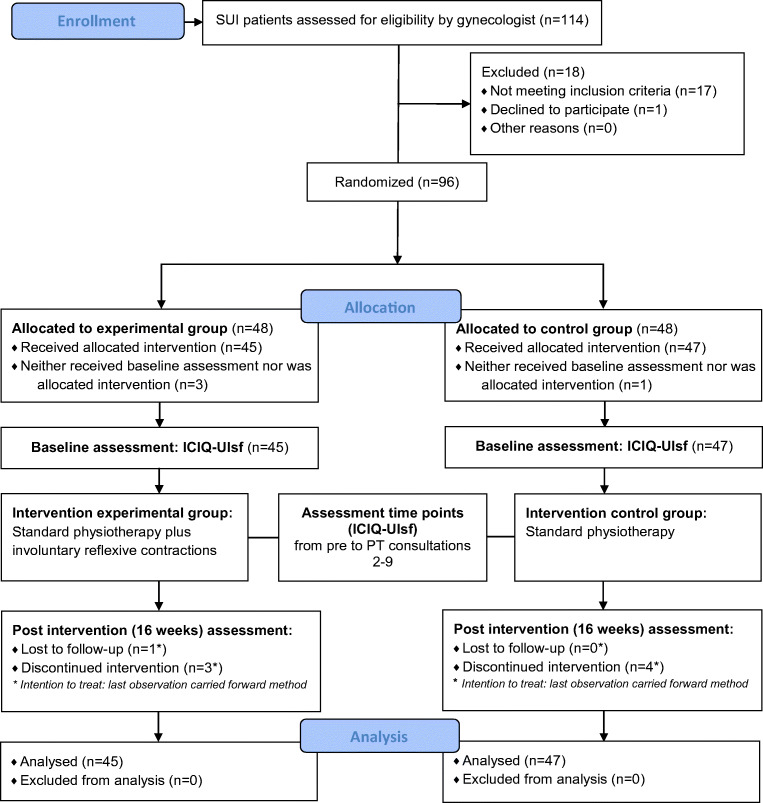
Consolidated Standards Of Reporting Trials study flow diagram. *SUI* stress urinary incontinence, *ICIQ-UIsf* International Consultation on Incontinence Modular Questionnaire Urinary Incontinence short form, *PT* physiotherapy

Figure 1 shows the flow of the participants through the study. During assessment of eligibility (*n* = 114), 18 participants had to be excluded (not meeting inclusion criteria (*n* = 17), declining to participate (*n* = 1)), resulting in 96 participants being randomized and allocated (EXP: *n* = 48; CON: *n* = 48). Subsequently, baseline assessment of 45 (EXP) and 47 (CON) participants was completed, as 4 participants were lost after randomization (EXP: due to personal reasons *n* = 1, medical problems *n* = 2; CON: not available *n* = 1).

Furthermore, 8 participants had incomplete primary outcome data (EXP: lost to follow-up post measurement (*n* = 1, discontinued intervention *n* = 3; CON: discontinued intervention *n* = 4). Based on the intention-to-treat approach and the applied last observation carried forward method, missing data were replaced by the last valid data of the respective participant (EXP: 4 participants with 1, 3, 6, and 7 missing data; CON: 4 participants with 5, 5, 7, and 8 missing data).

Regarding baseline characteristics in the two groups, there were no significant differences as to body measurements, number of births, and adherence to therapy schedule (Table 1). The latter was fulfilled to a high percentage by both groups (EXP, CON): home training sessions (93.4%, 87.7%), personal PT consultations (97.8%, 92.2%), and in total (94.3%, 88.2%).

**Table 1:** Demographics in the groups

Variable	CON , mean (SD)	EXP, mean (SD)	Significance, *p* value*
Participants, *n*	47	45	–
Age, years	52.3 (9.0)	50.5 (10.8)	0.395
Height, m	1.65 (0.06)	166 (0.06)	0.894
Weight, kg	69.7 (12.8)	66.8 (9.9)	0.242
BMI, kg/m^2^	25.3 (4.3)	24.2 (3.2)	0.176
Births (vaginal), *n*	1.6 (1.2)	1.7 (1.0)	0.661
Births (section), *n*	0.4 (0.8)	0.2 (0.6)	0.096
Births (total), *n*	2.0 (1.2)	1.9 (1.0)	0.699
Adherence (home sessions), *n*/*N*	68.4 (16.9)/78	73.3 (8.1)/78	0.081
Adherence (PT consultations), *n*/*N*	8.3 (2.0)/9	8.8 (1.1)/9	0.173
Adherence (total), *n*/*N*	76.7 (18.7)/87	82.0 (9.1)/87	0.085

*CON* control group, *EXP* experimental group, *SD* standard deviation, *n* absolute frequency, *N* total absolute frequency, *PT* physiotherapy

*Independent sample *t* test

### Primary outcome differences between the CON and EXP groups

**Fig. 2:**
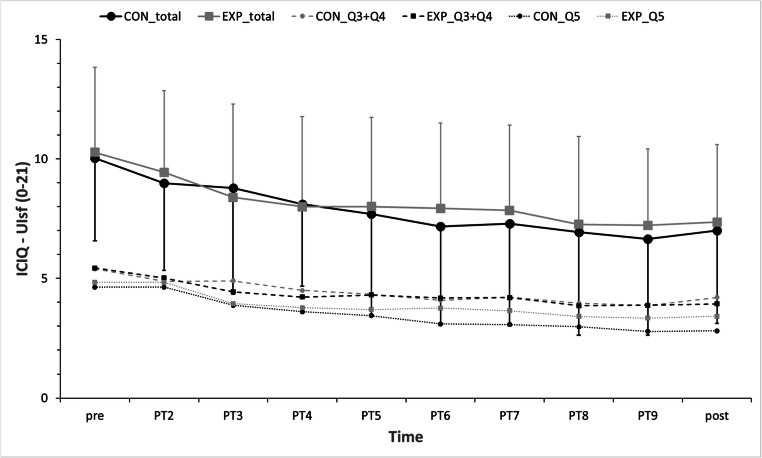
Mean and standard deviations of the control group and the experimental group of the total score of the International Consultation on Incontinence Modular Questionnaire Urinary Incontinence short form (ICIQ-UIsf) and means of its two subscales (Q3 + Q4 and Q5) during the ten measurement times from before to after the intervention phase. *CON* control group, *EXP* experimental group, *PT2…9* physiotherapy consultations 2…9

The mixed effect regression analysis (Fig. 2) did not show any differences (lower part of Table 2: Estimate [Group = EXP] * [Time = 1: pre … Time = 10: post]) between the EXP and CON groups for ICIQ-UIsf. Type III F tests for fixed effects were not significant at a 5% level, for neither the main effects of the groups nor the interaction effects between the groups and measurement times, for the total score of ICIQ-UIsf and its Q3 + Q4 and Q5 subscales.

**Table 2:** Estimates of fixed effect coefficients over 10 measurement times (time 1: pre … time 10: post) regarding total score, subscale Q3 + 4 and subscale Q5 of the International Consultation on Incontinence Modular Questionnaire Urinary Incontinence short form (ICIQ-UIsf) and for both groups

Dependent variable	Total score ICIQ-UIsf			Subscale Q3 + Q4 ICIQ-UIsf			Subscale Q5 ICIQ-UIsf		
Parameter	Estimate	Standard error	Significance	Estimate	Standard error	Significance	Estimate	Standard error	Significance
Intercept	7.01	0.54	<0.001	4.20	0.27	<0.001	2.81	0.34	<0.001
[Time = 1: pre]	3.03	0.68	<0.001	1.20	0.36	0.001	1.83	0.42	<0.001
[Time = 2: PT2]	1.98	0.66	0.003	0.68	0.35	0.054	1.30	0.40	0.001
[Time = 3: PT3]	1.77	0.64	0.006	0.70	0.35	0.043	1.06	0.39	0.006
[Time = 4: PT4]	1.10	0.61	0.073	0.29	0.34	0.376	0.80	0.37	0.032
[Time = 5: PT5]	0.69	0.58	0.231	0.14	0.32	0.669	0.64	0.35	0.069
[Time = 6: PT6]	0.17	0.54	0.751	−0.12	0.31	0.701	0.29	0.32	0.375
[Time = 7: PT7]	0.27	0.48	0.566	0.11	0.28	0.970	0.27	0.29	0.359
[Time = 8: PT8]	−0.07	0.41	0.856	−0.24	0.24	0.313	0.17	0.25	0.488
[Time = 9: PT9]	−0.36	0.30	0.230	−0.34	0.18	0.063	−0.02	0.18	0.906
[Time = 10: post]	0.00	0.00		0.00	0.00		0.00	0.00	
[Group = EXP]	0.34	0.77	0.656	−0.27	0.38	0.479	0.61	0.49	0.212
[Group = CON]	0.00	0.00		0.00	0.00		0.00	0.00	
[Group = EXP] * [Time = 1: pre]	−0.11	0.97	0.910	0.30	0.51	0.561	−0.41	0.59	0.493
[Group = EXP] * [Time = 2: PT2]	0.11	0.94	0.907	0.41	0.50	0.419	−0.30	0.58	0.606
[Group = EXP] * [Time = 3: PT3]	−0.73	0.91	0.421	−0.19	0.49	0.699	−0.54	0.55	0.331
[Group = EXP] * [Time = 4: PT4]	−0.45	0.87	0.605	−0.01	0.48	0.985	−0.44	0.53	0.405
[Group = EXP] * [Time = 5: PT5]	−0.04	0.82	0.965	0.24	0.46	0.604	−0.36	0.50	0.472
[Group = EXP] * [Time = 6: PT6]	0.41	0.77	0.595	0.36	0.44	0.408	0.05	0.46	0.921
[Group = EXP] * [Time = 7: PT7]	0.21	0.69	0.758	0.26	0.40	0.522	−0.04	0.41	0.916
[Group = EXP] * [Time = 8: PT8]	−0.01	0.58	0.980	0.17	0.35	0.630	−0.18	0.35	0.605
[Group = EXP] * [Time = 9: PT9]	0.23	0.43	0.596	0.28	0.26	0.276	−0.06	0.26	0.826
[Group = EXP] * [Time = 10: post]	0.00	0.00		0.00	0.00		0.00	0.00	

### Primary outcome differences across measurement times

The mixed model analysis of intervention effects (Fig. 2, Table 2) revealed that the average total score of ICIQ-UIsf decreased significantly between Time = 1: pre and Time = 10: post by about 3 points for both groups.

### Reaching the bottom level of primary outcome

For both groups the average total score of ICIQ-UIsf (Fig. 2, Table 3) differed significantly at a 5% level from time = 10: post at time = 1: pre and time = 3: PT2 according to estimated mixed models. In other words, both groups reached the bottom level at time = 4: PT3. For the ICIQ-UIsf subscales the EXP group reached the bottom level at time = 4: PT3 and the CON group at about time = 6: PT5.

**Table 3:** Estimates of International Consultation on Incontinence Modular Questionnaire Urinary Incontinence short form (ICIQ-UIsf) differences between the level at measurement times 1 (pre) to 9 (PT9) and the level at measurement time 10 (post), separately for each group. Note that the estimates for the control group can also be found in Table 2

	Total score ICIQ-UIsf		Subscale Q3 + Q4 ICIQ-UIsf		Subscale Q5 ICIQ-UIsf	
	CON	EXP	CON	EXP	CON	EXP
[Time = 1: pre]	*3.03*	*2.92*	*1.20*	*1.50*	*1.83*	*1.42*
[Time = 2: PT2]	*1.98*	*2.09*	*0.68*	1.09	*1.30*	*1.00*
[Time = 3: PT3]	1.77	1.03	0.70	0.51	*1.06*	0.52
[Time = 4: PT4]	1.10	0.64	*0.30*	0.29	*0.80*	0.36
[Time = 5: PT5]	0.69	0.66	0.14	0.38	0.64	0.28
[Time = 6: PT6]	0.17	0.58	−0.12	0.24	0.29	0.33
[Time = 7: PT7]	0.27	0.49	0.01	0.27	0.27	0.22
[Time = 8: PT8]	−0.07	−0.09	−0.24	−0.08	0.17	−0.01
[Time = 9: PT9]	−0.36	−0.13	−0.34	−0.06	−0.02	−0.08
[Time = 10: post]	0.00	0.00	0.00	0.00	0.00	0.00

Numbers in italics refer to significant parameters (significance ≤0.05)

### Comparisons of secondary outcomes

All pre−/post within-group comparisons (20-min pad test, muscle strength test, and ICIQ-LUTSqol part B) showed a significant improvement, except for EXP ICIQ-LUTSqol part A (EXP: 38 to 38 points, >0.001; CON: 39 to 36 points, <0.001). There were also no statistically significant differences at any time between the groups, except in muscle strength (pre; Table 4).

**Table 4:** Results of pad test, manual muscle testing (modified Oxford Grading Scale), and quality of life (LUTSqol: part A, part B)

Variable		CON, mean ± SD or median (IQR)	EXP, mean ± SD or median (IQR)	Significance, *p* value (between)
Pad test, g	Pre	12.5 ± 21.0	10.8 ± 19.3	0.690***
	Post	5.1 ± 9.2	5.7 ± 16.0	0.818***
	*p* value (within)*	*0.005*	*0.008*	
Oxford (0–5)	Pre	3.0 (1.0)	2.3 (1.3)	0.007****
	Post	3.3 (1.3)	3.0 (1.3)	0.262****
	*p* value (within)**	*0.019*	*<0.001*	
LUTSqol (part A)	Pre	39 (17)	38 (15)	0.474****
	Post	36 (14)	38 (14.5)	0.576****
	*p* value (within)**	*0.007*	0.055	
LUTSqol (part B)	Pre	48 (49)	52 (51.5)	0.351****
	Post	20 (43)	29 (44.5)	0.425****
	*p* value (within)**	*<0.001*	*<0.001*	

Numbers in italics refer to significant parameters (significance ≤0.05)

*CON* control group, *EXP* experimental group, *SD* standard deviation, *IQR* interquartile range

*Dependent sample *t* test

**Wilcoxon signed rank test

***Independent sample *t* test

****Mann–Whitney *U* test

The treating physiotherapist asked the participants if urinary loss had occurred during the two training sessions at the personal PT consultations. None of the women had urinary loss during the exercises (jumping/running on the spot).

## Discussion

The analysis of two newly developed PFMT protocols, one including standard PFMT and one additionally focusing on involuntary reflexive PFMT, regarding their intervention effects on SUI revealed that the total score of the primary outcome ICIQ-UIsf decreased significantly over time by about 3 points for both groups. However, contrary to the hypothesis, there were no differences between groups at any timepoint. The same applies for the secondary outcomes (pad test, PFM strength test, quality of life). Exceptions were ICIQ-LUTSqol part A, with the CON group showing significant improvement, the EXP group being only close to significance, and the PFM strength indicating a significant difference at baseline (CON > EXP).

Adherence was equally as high in the two groups for personal PT consultations as well as home training sessions.

These results mean that the present training protocol of the experimental group, which additionally focused on involuntary reflexive PFMT, could not show any additional benefit compared with standard PFMT regarding the treatment of stress urinary incontinence.

Interestingly, ICIQ-UIsf score improvement had already reached a floor effect at PT 3 (after 4 of the 16 intervention weeks) in both groups. Because in the first 4 weeks basic information was provided and instructions were practiced in addition to PFMT (information regarding anatomical and (patho-)physiological aspects of SUI, explanation of the function of the pelvic floor, interaction between diaphragm and PFM) [16], it is not possible to draw conclusions about whether the PT effect was due to PFMT or basic information and instructions.

In a comparable study population (baseline mean age, mean ICIQ-UIsf scores and pre- to post-measurement time points) Nyström et al. [21] found that a change in ICIQ-UIsf of ≥2.52 of 21 scores reflected clinically relevant improvements after PFMT in women with SUI. Hence, the current study showed clinically relevant improvements (about 3 points) in SUI in the CON group as well as in the EXP group. After the intervention, both groups still reported moderate SUI (about 7 points) according to the definition of an ICIQ-UIsf score of 6–12 [22], meaning that the participants were still leaking and not completely continent.

Riemsma et al. [23] performed an extensive systematic review on cure rates of incontinence after various interventions, such as surgery, PT, medication, etc. As for SUI, cure rates with supervised PFMT ranged from 5% to 74.8%. However, the studies included used different definitions of cure such as “completely dry,” “a negative cough stress test,” or “much better or very much better on Patient Global Impression of Improvement,” which indicates that the study participants were not necessarily completely free from SUI after the respective intervention and makes it difficult to compare studies. Dumoulin et al. [13] compared PFMT for women with SUI with no treatment, placebo or sham treatments, or other inactive control treatments. Besides studies with other outcomes, they also retrieved several articles using ICIQ-UIsf as an outcome. None of those studies ended in complete absence of SUI after the intervention, and the improvement in ICIQ-UIsf score number was comparable with the results of the present study [13].

Even though PFMT, defined as exercise to improve PFM strength, endurance, power, relaxation, or a combination of these parameters [24], has been shown to be effective [13], the question arises why PFMT cannot restore SUI to a higher percentage than that found by Riemsma et al. [23]. Possible reasons for “only” improvement but often not “complete” cure of SUI could be that relevant training methods (for hypertrophy [improving muscle mass], intramuscular coordination [improving synchronous muscle fiber recruitment and innervation frequency], power [improving rate of activity], and power endurance [improving endurance of rate of activity]) are often not clearly and consistently defined regarding their training parameters [25] and not strictly implemented as progression phases in PFMT protocols. Also, current guidelines for SUI with related PFMT do not or barely mention them [12, 26, 27]. Additionally, those training methods cannot be applied as easily to the PFM as to other skeletal muscles: challenges of the transferability of common training methods to the PFM are, for example, maximum strength and hypertrophy training demanding the use of external weights, high loads, and fatigue [14], PFM anatomy and location allowing only for a tiny range of motion [28], and scarce evidence regarding the effect of specific training methods on the PFMs. To the authors’ knowledge there is only one study that tested for PFM morphology (thickness, levator hiatus area, and pubovisceral muscle length) in women with pelvic organ prolapse before and after PFMT, which has been proved effective for women suffering from SUI [29]. If one compares the effect regarding the increase in muscle mass (15.6% relative to baseline) of this study with that of other studies, significantly higher increases in muscle mass of 22–37% in the leg muscles, for example, can be seen [30].

Compared with common skeletal muscle training the following points could have impaired the outcome of the present trial and therefore are a limitation of the interpretation of its results:

1. For feasibility reasons training methods were applied during rather too short time intervals (4 months in total) compared with scientifically based and common phase durations of skeletal muscle training method phases (at least 6 months in total), which are necessary because of adequate periods of biological adaptation (sensorimotor components, inter- and intramuscular coordination, hypertrophy, etc.) [14]. In particular, this could be a major reason why the EXP group no longer made any progress in these training phases. This resulted from a pragmatic clinical trial approach, in terms of therapy duration, to be comparable with common SUI PT (9 personal PT consultations = typical PT duration according to medical prescription and meeting of costs by health care insurance in Switzerland). Other reasons were recruitment and participation issues such as manageable therapy and study time span for participants.
2. Training standardization as necessary for an RCT and an approach according to scientific criteria prohibit adaptation of the training parameters to individual factors and progress status to guarantee comparability. Individual training method phase length would need specific criteria regarding functional perception and movement quality, muscle mass, strength, power, and power endurance of the PFMs for start and termination of each progression phase of training. Hodges et al. [31] introduced such criteria for PFM motor learning—coordinative training to improve a movement sequence done by improving inter- and intramuscular coordination—for men after radical prostatectomy, as so far, those and the other above-mentioned criteria for the phase lengths of training methods (i.e., criteria defining when to start and when to terminate a specific training method phase and therefore indicating its time period) are non-existent in PFMT for women with SUI.
3. Compared with common skeletal muscle training [14], the training methods for hypertrophy, intramuscular coordination, power, and power endurance might not have been applied consistently enough regarding intensity, exhaustion, and strain for feasibility reasons (e.g., no external weights). As nothing is known about the improvement of power and power endurance of the PFMs so far, the known training methodology that was applied in the EXP group would have to be investigated specifically for the PFMs and modified if necessary.
4. A specific study limitation was that in the EXP group, a few participants could for a short time period not perform exercises of involuntary reflexive PFMT such as running on the spot or jumping owing to, for example, knee or low back complaints, or fear of jumping because of perceived lack of physical fitness. The reasons were therefore neither related to the training protocol of the EXP group nor caused by these specific training exercises.
5. A very fundamental question is how well participants with an initial maximum strength of an Oxford grade M3 or lower can be trained at all. With such severely weakened PFM strength, it is possible that the maximum strength would first have to be increased (e.g., at least M4) in order to achieve effects in terms of hypertrophy and power. Additionally, it has to be taken into account that the EXP group had a significantly lower PFM strength at the beginning of the study. This could have limited the possible effects of the EXP group in comparison with the CON group.

A potential weakness of the present trial is that reflex activity and neural components were not assessed initially, which would have probably been feasible by pudendal nerve latency measurement. However, this technique is rather difficult to apply in our setting and frequent false-positive results may be achieved [32].

The aim of this first study with additional reactive PFM training was to fundamentally evaluate whether or not an additional effect could be shown. Therefore, and for reasons of recruitment and feasibility, the inclusion criteria were relatively broad. Even though this was a randomized controlled trial and no differences regarding baseline characteristics were shown between the groups, the rather broad inclusion criteria, such as large age span or number of births, could indicate a weakness of this trial. More specific samples should be chosen for future investigations.

Study strengths are the adequate statistical power, with few drop-outs and detailed standardized PT protocols in terms of basic information, instructions, and phases of PFMT methods for motor learning, strength, hypertrophy, power, and power endurance, with exact description of the training methodology such as muscle action, velocity of muscle action, loading, volume, rest periods, and frequency. Moreover, to the best of the authors’ knowledge, this trial is the first to investigate a PFMT protocol focusing on involuntary PFM reactivity, i.e., not only focusing on voluntary PFMT.

## Conclusion

This RCT showed a clinically relevant effect on SUI of two PT protocols—one including standard PT and one additionally focusing on involuntary reflexive PFM contractions. However, there was no difference regarding effect size between those two PFMT protocols, meaning that the PFMT protocol additionally focusing on involuntary reflexive PFM contractions—as was done in this trial—did not show any additional benefit regarding SUI. Future studies should use criteria-oriented and not time-oriented PFMT protocols, i.e., individualized PFMT protocols and progress. Therefore, criteria for goal obtainment of each PFMT method phase would have to be developed and defined.

The investigation of function-oriented PFMT methods for hypertrophy, intramuscular coordination, power, and power endurance training, which are comparable with “common” skeletal muscle training, i.e., performed with higher intensities and workout, as well as basic information and instructions versus only PFMT, should be investigated.
